# Gene Expression Profiles from Needle Biopsies Provide Useful Signatures of Non-Small Cell Lung Carcinomas

**Published:** 2007-06-08

**Authors:** Douglas E. Paull, Kevin Kelley, Jazbieh Moezzi, Madhavi Kadakia, Steven J. Berberich

**Affiliations:** 1 Departments of Surgery; 2 Biochemistry & Molecular Biology; 3 Pathology, and the; 4 Center of Genomics Research. Wright State University, Boonshoft School of Medicine, and VA Medical Center, Dayton, Ohio

**Keywords:** Gene expression profiles, Lung cancer, Needle biopsy, Staging

## Abstract

Gene expression profiles from DNA microarrays can provide molecular signatures that improve tumor classification, prognosis, and treatment options. While much of this work has focused on isolation of RNA from the resected tumor, fewer studies have utilized RNA from fine needle aspirates (FNA). In this pilot study we examined whether the gene signatures obtained from FNA samples would correlate with signatures taken from the resected tumor. Based on NSCLC gene expression profiles obtained from eleven sets of FNA and tumor samples we obtained a high concordance of FNA profiles matching their matched tumor sample. These results suggest that FNA samples may provide informative gene expression signatures regarding the potential aggressiveness of non-small-cell lung carcinomas.

## Introduction

Lung cancer is the most common cause of cancer death in the U.S. (Socinski, Schell et al. 2002). Non-small cell lung carcinomas (NSCLC) account for 70–80% of lung malignancy (Fu, Zhu et al. 1999). The majority of patients with NSCLC present with advanced stage III or IV disease. Despite advances in the diagnosis, staging, and treatment of NSCLC, the overall 5-year survival rate remains poor (Sause, Kolesar et al. 2000).

In an effort to improve the treatment of NSCLC, molecular “fingerprinting” using DNA microarrays, has been undertaken (Nacht, Dracheva et al. 2001). This technology has led to the identification of gene expression patterns that are improving NSCLC tumor classification, biomarker identification, prognosis/outcome predictions, understanding of altered molecular pathways, and targeted therapies (Petty, Nicolson et al. 2004).

Classical gene expression profiling (GEP) requires a tissue sample from the tumor, RNA extraction, and DNA hybridization to determine which genes are expressed in a given tumor. While GEP has proven useful in lung cancer patients, samples have generally been derived from surgical specimens in patients with resectable disease (Beer, Kardia et al. 2002). Unfortunately, most patients with NSCLC have unresectable disease. Further, in the future, even patients with resectable disease may benefit from neoadjuvant therapy prior to resection. In both of these situations, GEP data derived from needle biopsy would be potentially beneficial.

The few studies examining GEP from fine needle aspirations (FNAs) of lung cancer have been disappointing, in part due to RNA degradation (Lim, Aggarwal et al. 2003). The purpose of this pilot study was to compare GEP profiles from needle biopsies to GEP profiles from the resected tumor in the same cohort of patients.

## Materials and Methods

### Sample collection

The study was approved by the Institutional Review Boards of Wright State University School of Medicine and the Dayton VA Medical Center on Dec. 17, 2004. After informed consent, a total of 14 patients with suspected resectable non-small cell lung cancer (NSCLC) entered the study between Oct. 4, 2005 and May 16, 2006. Two of the 14 patients ultimately were found not to have NSCLC on final pathologic analysis (one lymphoma, one metastatic renal cell carcinoma), resulting in a study of 12 patients with NSCLC. One additional needle sample (adenocarcinoma) had to be excluded from the analysis due to poor array data resulting in a final 11 sets of tumor and needle gene expression profiles (Table 1).

### Patient treatment

Patients underwent an extensive preoperative history, physical examination, and radiologic and laboratory testing. Studies included pulmonary function testing, arterial blood gas analysis, cardiac stress testing, CT scans of the chest, positron emission tomography (PET) scans, bronchoscopy, and mediastinoscopy in an effort to preoperatively stage the patient and determine perioperative risk. Several of these indices can be found in Table 2.

At operation, all patients underwent bronchoscopy to determine proper position of double-lumen endotracheal tubes. Prophylactic antibiotics were given. Patients either underwent video thoracosopy or posterolateral thoracotomy for initial wedge resection or biopsy of the lesion. During the operation, just prior to resection of the lung mass, a true cut needle biopsy with an 18G detachable biopsy system (Boston Scientific, Natick, MA) was performed either thorascopically or via thoracotomy. Specimens were immediately placed in RNALater solution (Ambion, Austin, TX). An additional needle biopsy specimen was sent for frozen section. Once malignancy was confirmed, the lesion was resected via wedge resection, lobectomy, or pneumonectomy depending on the size and location of the tumor and the health of the patient. Additional large specimens from the resected (or open biopsied) parent tumor were also placed in RNALater solution. Following resection, a systematic mediastinal lymph node sampling was performed. Immediately postoperatively, needle biopsy and resected specimens were transported to the Wright State Univ. Center for Genomics Research (WSU-CGR).

Patients were extubated in the operating room or recovery room. Pain management postoperatively included an epidural catheter infusion of fentanyl and bupivicaine. Complications were defined according to a previous publication from this Department (Pezzella, Adebonojo et al. 2000). Charlson comorbidity scores were calculated (Birim, Zuydendorp et al. 2003). Statistical analysis was performed using the NCSS/PASS 2000 (NCSS Statistical Software, Kaysville, UT) and InStat (GraphPad, San Diego, CA) software programs. Survivorship was determined using the Kaplan-Meier product limit method.

### RNA isolation

Tumor and needle biopsy samples were pelleted, RNAlater removed and tissues stored at −80 °C until the samples were processed. Frozen tissues were placed in liquid nitrogen and then transferred to a tissue pulverizer pre-frozen overnight at −20 °C. The pulverized tissue was resuspended in Trizol reagent and total RNA isolated following manufacturer’s recommendations (Invitrogen, Gaithersburg, MD). An aliquot of the eluted RNA was analyzed using a RNA Nano 6000 chip on an Agilent Bioanalyzer (Agilent, Santa Clara, CA). For each sample, generation of biotinylated cRNA began with six hundred nanograms of total RNA employing a two-step Affymetrix labeling kit as described by the manufacturer (Affymetrix, Santa Clara, CA).

### DNA microarray

Fragmented biotinylated cRNA probe was hybridized to an Affymetrix GeneChip HGU133 Plus 2 containing over 47,000 putative gene transcripts. The GeneChips were incubated for 16 hours at 42 °C prior to washing, staining and scanning following Affymetrix protocols.

### Data analysis

Affymetrix GeneChip CHP files, scaled to 150, were loaded into GeneSpring 7.3 for data analysis. For studies examining all 22 GeneChips (needle and tumor) a genelist consisting of genes present (P) in at least 1 out of 22 GeneChips was created (42093 genes). When determining if the gene expression profiles of needle biopsies mirrored their tumor biopsy match a condition tree was run using a Pearson correlation and average linkage using the 42093 genes that were present in one of the 22 GeneChips used in the analysis linkage. Identical results were obtained when the analysis was run using only genes present in all 22 samples (data not shown). Bootstrapping (1000 iterations) was performed to derive a confidence value for each branch of the condition tree. All FNA:Tumor matches had 100% confidence values.

## Results

Since most patients with non-small cell lung carcinoma are diagnosed with advanced disease, resection may not be an option. In this study we wanted to examine whether needle biopsies would provide gene expression profiles that matched those obtained from resected tumors. While a prior study suggested fine needle aspirates (FNA) produced expression profiles that matched their respective tumor samples; only 40% of the FNA samples provided RNA of sufficient quality for analysis (Lim, Aggarwal et al. 2003). In this study we recruited 14 patients over a six-month period from which 12 patients were diagnosed with NSCLC. Table 1 contains the information regarding these patients’ tumor type, yield of RNA, and quality of the RNA based on RIN analysis using a RNA nano 6000 chip (Agilent Bioanalyzer). As expected we recovered only a fraction of RNA from needle biopsies compared to tumor biopsies (0.7–26%, Table 1). Nevertheless, the quality of the RNA as determined using the RNA integrity (RIN) values (Schroeder, Mueller et al. 2006) was above average for both types of samples.

**Figure 1:**
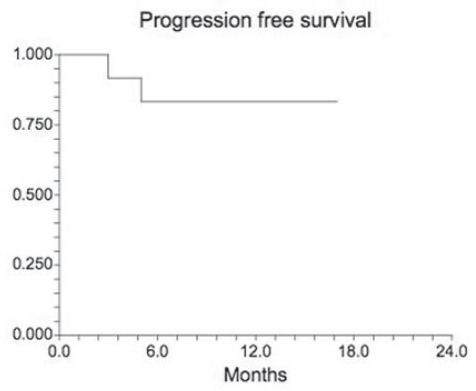
Kaplan- Meier survival plot. Survival of the 11 patients in this study. Only two individuals have died during follow-up, consistent with the observation that most of these tumors were early stage NSCLCs (Table 1).

The majority of patients were smokers with moderately severe emphysema by pulmonary function testing (Table 2). With the exception of their pulmonary disease, patients were in otherwise reasonable health as judged by high performance status and low comorbidity indices. Most patients had early stage disease, and had good long term survival, consistent with a study of “resectable” lung cancer (Figure 1). The predominance of squamous cell histology is due to predominantly male, heavy smoking patients in a VA population as previously reported (Paull, Updyke et al. 2005).

**Figure 2:**
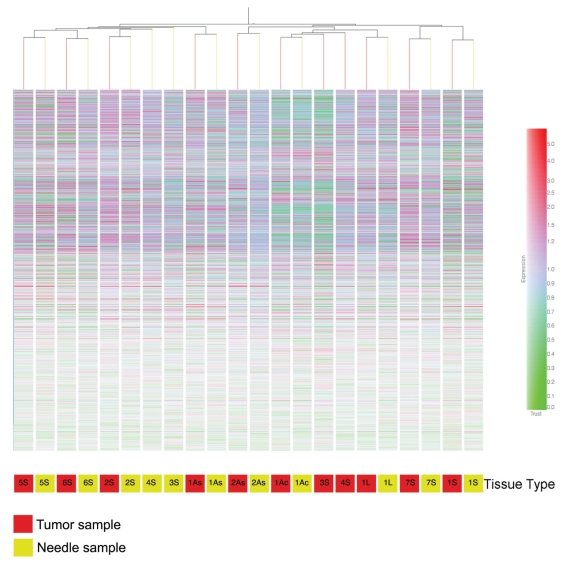
Condition Tree of the 22 GeneChip profiles compared based on tissue type. The 11 sets of gene expression profiles were compared using the Pearson correlation with average linkage. The tree details the relationship of the 22 profiles with the gene signals (red-high expression, green-low expression) shown for each of the 42093 genes that were analyzed. Of the 11 sets of profiles, 9 of them showed that the FNA profile was most similar to the profile from its matching tumor. Colorbar represents how the color changes relate to relative expression.

To ascertain whether the FNA samples contained a sufficient amount of tumor sample to provide a gene expression profile indicative of the tumor sample, the gene expression profiles from the 11 needle aspirates were compared to the 11 tumor biopsies using a Pearson correlation coefficient. As shown in Figure 2, nine of the eleven needle samples (81.8%) were most like their tumor match (Figure 2). Only FNAs 3S and 4S, both from squamous cell carcinomas, failed to correlate with their tumor samples. Through the use of bootstrapping we were able to demonstrate that these matches (FNA:Tumor) could not occur by chance.

**Figure 3:**
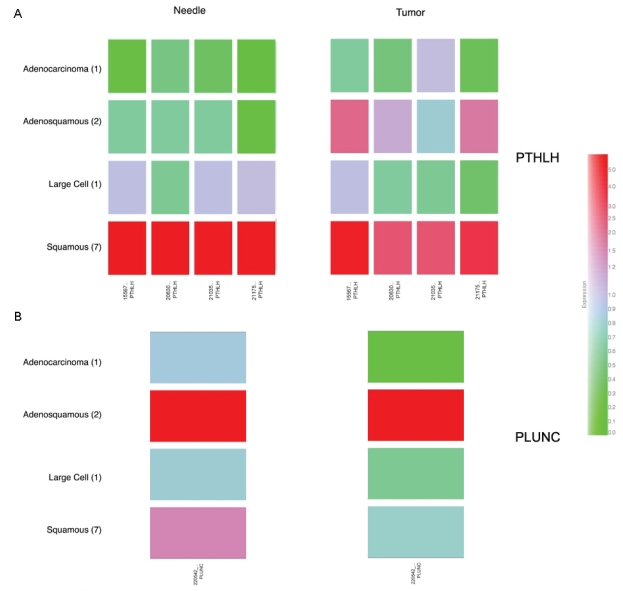
Examination of PTHLH and PLUNC relative expression across various histological NSCLCs. The 11 needle and 11 tumor profiles were grouped into one of four histological subtypes and gene expression levels averaged among each group. (A) PTHLH expression is high in both the needle and tumor squamous samples (n = 7) and lowest in the adenocarcinoma (n = 1) and adenosquamous samples (n = 2). All four probesets for PTHLH show similar expression patterns. (B) PLUNC probe set shows that highest expression in the adenocarcinoma samples (n = 2) and is also detected in the squamous cell needle and tumor samples (n = 7). The expression of PLUNC appears higher in needle samples compared to tumor samples in the adenocarcinoma and large cell profile. This may result from the fact that the needle biopsies contain some normal tissue and PLUNC is found expressed in normal lung tissue (Bingle, Cross et al. 2005). Colorbar represents how the color changes relate to relative expression.

In addition to using this unbiased approach, we also mined the microarray data using genes known to be altered in NSCLC. Parathyroid hormone like hormone (PTHLH) has been previously identified as being induced in NSCLCs (Nishigaki, Ohsaki et al. 1999; Hiraki, Ueoka et al. 2002). After grouping the needle and tumor biopsy array data by tumor type we confirmed that PTHLH was indeed induced, most strongly in the squamous cell carcinoma tissues (Figure 3A). Another potential target gene PLUNC was originally identified by differential display as being detected in 80% of NSCLC tumors that were lymph node positive (Iwao, Watanabe et al. 2001). Although the Affymetrix GeneChip only possesses a single probe set targeting PLUNC, we found the gene expressed in all tumors examined with elevated expression in the adenocarcinoma (Figure 3B). Taken together these results from the unsupervised hierarchical clustering approach (Figure 2) and examination of genes known to be altered in NSCLC (Figure 3) suggest that the FNA gene profiles were a good representation of the tumor gene expression profile.

## Discussion

The goal of this pilot project was to determine whether high quality RNA could be isolated from fine needle aspirates taken from patients with non small cell lung carcinomas and if so how well the gene expression profiles obtained from these specimens represented the gene expression profiles from the actual tumors. Unlike a previous study (Lim, Aggarwal et al. 2003), we obtained high quality RNA from FNAs (Table 1) and even though the yields for several samples were rather low, were able to employ the Affymetrix Two-Step cDNA labeling procedure to produce a sufficient amount of biotinylated cRNA to label HGU133 Plus 2 GeneChips. Based on a statistical comparison of the GEPs from the FNAs and tumor samples over 80% of the FNAs GEPs most closely correlated with their matching tumor sample. It is possible that the GEPs from the two FNAs which did not match their respective tumor sample may have resulted from a higher than acceptable amount of non-tumor RNA within the FNA. Future studies to correlate matches with the percentage of tumor tissue within a FNA will provide an important criteria to enable clinicians to assess whether a given FNA is suitable for microarray analysis. The finding of concordance between GEP profiles obtained from needle biopsies and the parent tumor for patients with non-small cell lung cancer is significant. Up to this point, only patients with early stage, resectable lung cancer were in a position to be aided by GEP data (Beer, Kardia et al. 2002). Unfortunately, patients with early stage lung cancer make up only the minority of all lung cancer patients. Needle biopsy derived GEPs would allow patients with unresectable lung cancer to derive potential benefits; for example, prognostic information, chemotherapy selection, or targeted therapies (Petty, Nicolson et al. 2004). In addition, it is likely that in the future, even patients with resectable lung cancer will undergo neoadjuvant chemotherapy prior to resection (Patel, Blum et al. 2004). Needle biopsy GEPs could conceivably play a role in such a setting. These results suggest that obtaining microarray results from FNAs is feasible, provides data that is representative of the primary tumor and may provide gene signatures of early stage NSCLCs that can be used to predict outcomes.

## Figures and Tables

**Table 1:** Tumor Histology and RNA Quantifications.

Code	Tumor Type	RNA Yields (T/N; μg)	RIN Values (T/N)
1S	Squamous	1589/10.8	NA
1L	Large Cell	414/5.8	NA
2S	Squamous	233/17.3	8.9/9.2
3S	Squamous	232/14.4	7.2/9.2
4S	Squamous	79/21	8.8/9.4
5S	Squamous	369/22	8.3/9.7
1As	Adenosquamous	34/0.7	9.2/8.9
2As	Adenosquamous	154/12.5	7.4/8.7
6S	Squamous	131/14.6	9.2/8.9
1Ac	Adenocarcinoma	36.5/2.6	7.3/7.5
7S	Squamous	211.5/29.5	9.4/8.4

**Table 2:** Patient Characteristics.

Variable	Result
Age (years)	61.9 ± 3.1
Sex (males/females)	11/0
Pack years smoking	64.1 ± 12.4
Current smoker	7/11 (64%)
Charlson comorbidity index	1.8 ± 0.3
Symptoms	9/11 (82%)
Dyspnea	6/11 (55%)
Weight loss	2/11 (17%)
ECOG performance status	0.8 ± 0.2
Karnofsky performance status (%)	87 ± 3
PO_2_ (mm Hg)	82.4 ± 5.1
PCO_2_ (mm Hg)	39.0 ± 1.0
FEV1 (liters)	2.39 ± 0.2
FEV1% predicted	69.8 ± 5.9
DLCO% predicted	49.7 ± 4.7
Operation	
Lobectomy	7611 (55%)
Wedge resection	3/11 (27%)
Pneumonectomy	1/11 (9%)
Biopsy only	1/11 (9%)
Tumor size (cm)	4.6 ± 0.6
Stage	
I	7/11 (64%)
II	3/11 (27%)
III	1/11 (9%)
Complications	2/11 (18%)
Perioperative death	0/11 (0%)
Chemotherapy	10/11 (91%)
Radiation	4/11 (36%)
